# Dentistry and Physic

**Published:** 1845-03

**Authors:** 


### ARTICLE III.

### *Dentistry and Physic.* By the SYRACUSE EDITOR.

A FEW weeks since, a gentleman (if we may be pardoned for thus using the term) called on us, and introduced himself as Dr. ——, from an adjoining town, and inquired if we kept instruments to sell. Having repeatedly had similar calls, we readily anticipated to what class and order he belonged. We inquired, in turn, if he practised dentistry, to which he replied, "no, not much; I am only intending to do a few *simple operations*, such as *cleaning, filling, and setting pivot-teeth.*" Having a curiosity, at least, to know something of his proficiency in these "*simple operations*," we asked him to step to our operating table, and select such instruments as he wished; adding, that we could not, perhaps, spare such as he might desire. He met this proposition by remarking, that he "did not exactly know what kind of instruments he did want, as he had not as yet done any thing at the business, and he had called on" us "because he presumed" *we* "should know what would be necessary."

After very frankly expressing to him our own views as to the *simplicity* of these operations, and the effect which similar attempts had produced upon the public mind, in lowering the true standard of the dental profession, we informed him that though we had facilities for making our own instruments, and uniformly did so, with very few exceptions, we never yet sold one, and that we certainly could *not* consent, under any circumstances, to sell instruments to any person, if he intended to *use* them, so ignorant as not to know which to select for any given purpose. Though our remarks seemed to dampen somewhat his ardor, we subsequently learned from one of his patients, who exhibited a specimen of his operations, that they did not deter him from his anticipated undertaking; adopting, we have no doubt, the motto, that "there is nothing like trying;" reminding us of the man, who, upon being asked if he could read *Greek*, replied that he did not know but he could, as he had never tried.

The specimen we saw, and we hope the only one we shall ever have the *pain* of seeing, of the skill of this new adventurer, was a case of plugging, or rather *stuffing*, two adjacent cavities, by one operation; the gold being pressed between the teeth, filling the entire space, which disease, and his otherwise clumsy operation, had made. Were this a solitary example of these amphibious practitioners, we should by no means deem it worthy of record, but when we reflect that it is quite common for young physicians, and, sometimes, older ones, especially in small villages, to keep a few instruments, or, as we might better say, tools, (if awls and the like should be thus ranked,) we feel that it is time some effort was directed to the removal of this great evil.

In offering some remarks upon this case as a *text*, we would call attention to those prominent sources of popular error.

1st. Those operations which are generally regarded difficult, and as sure tests of skill, and are, hence, made the basis of confidence, and commendation; are, in fact, the most simple, and may be performed by the merest quack.

2d. Those operations which are commonly esteemed the most simple, are by far the most difficult, and involve infinitely more skill than the former.

3d. Physicians receiving only a medical education, are capable of performing neither aright, and hence should never undertake them.

Perhaps, no fact is better understood, or more lamented, by all honorable men of the dental profession, than that the majority of persons lay far more stress, *as the measure of skill*, on specimens of artificial work, than upon any operations performed upon the natural teeth. How frequently do we hear an operator, and may be, a contemptible mountebank, lauded as a "splendid workman," not only by non-professional men, but by M. D's. and D. D's., each of whom may be regarded equally good judges, for a show specimen of plate-work, which the most ignorant mechanic may construct. It is a fact, worthy to be remembered, though too often overlooked, that no man, however learned, is a competent judge of skill in any art, or science, to which he has paid no particular attention. Nay, we have often seen the very elevation, which great condition has created, made the stumbling-block to the greatest imposition. Such men feeling that they are looked upon as possessed of extraordinary intelligence, do not presume that *any* would have the audacity to offer to *them* imposture; and, hence, that suspicion, which is on the alert in less cultivated minds, and which, in a measure, serves to protect them from imposition, being less active, they fall an easy prey to empiricism, and, too, strange as it may seem, by reason of the very influence, upon which they had relied for protection against such attacks. When, therefore, we consider the imposing exhibition which may be made of artificial work, especially as compared with operations upon the natural teeth, which every judicious dentist seeks as much as possible to hide from view, we need not wonder at the multiplicity of recommends obtained by the merest quack, even from those bearing, and, perhaps, meriting, high sounding titles. But such men would do a great service to humanity by remaining in silence upon those subjects upon which they have no knowledge.

They should remember, that upon *them* rests the responsibility, having the power to do great good, or great harm, by a distinction in other matters, awarded them by an entire community. But no matter how extensively the idea may have prevailed, that a beautiful piece of plate work should secure to the artist a just claim to reputation, and skill, as a surgeon dentist, no mistake is greater or more fatal to the best interests either of the profession, or the community at large. For, unless we include in the idea of plate-work, the skill required to determine *how* the operation is to be performed as regards the *mouth*, how many of the natural teeth, if any, may, and should be preserved there, the particular arrangement best calculated to secure the greatest benefit, and a multitude of other considerations often involving great experience, skill and judgment; we may regard this kind of work a test of nothing but a proficiency on the part of the artist in strictly mechanical acquirements, and this, too, in a branch of easy attainment; even by one who could neither read or write, and who might not be able to distinguish between the jaw of a man and a sheep.

Now the truth is, the recommends referred to, have, as a general rule, no reference whatever, to the considerations above alluded to as involving skill, nor could they have; but simply to the *work* wholly disconnected with the mouth. To offer, therefore, the ready opinion that a man is a skilful *surgeon dentist*, and have it based upon such data, is as preposterous, and as clearly ridiculous, as to affirm that the man who could construct a glass-eye, or a cork-leg, was a good oculist or an eminent surgeon. We not unfrequently see in the advertisements of *quacks*, and we believe it confined to such, special pains taken, to notify the community that they have arrived at so great a state of perfection, in dental surgery, even to be able to *manufacture* incorruptible teeth!! and this effort is by no means lost with a large portion of community. Now could such men give good evidence that they could make good stone-jugs, or a decent article of porcelain ware, they would give evidence equally conclusive of their skill in dental surgery, and far better proof that they were making themselves useful to their race. When we consider that the most beautiful piece of plate-work, may be, and often is made, by the jeweller, who has not the most distant idea of its adaptation, or what constitutes a healthy mouth, or any means to make it such, and that our most splendid porcelain-teeth are *generally* manufactured by men ignorant of almost every thing else, and even by girls, who, in many instances never knew the meaning of the word dentistry, we may clearly see the absurdity of necessarily ascribing skill as a dental surgeon, to those who have not only manufactured artificial teeth, but who have learned, also, the ennobling secret of setting them upon a gold plate. But while we thus speak of these operations, we would by no means wish to detract any thing from their real value. On the other hand, we consider the invention of porcelain-teeth one of the greatest acquisitions to dentistry, and the ability to construct artificial work, as absolutely essential, that the dentist may bestow the full benefit of his profession upon his patients. Nevertheless, we regard neither, as any proof of skill in *far the most important* of dental operations, to say nothing of their being no index to scientific attainments.

Let us now proceed to examine, hastily, the simplicity of those operations which Dr. ——, and far the greater majority of people regard "simple operations," and of such easy attainment. Our remarks, hitherto, have been directed to those operations, or rather *structures*, requiring only a knowledge of physical, and some practical knowledge of chemical laws, all of which are uniform in their operation and application, and are hardly subject to modification, and which are, therefore, so far as practice is concerned, of easy acquisition. We now, however, come into a very different field, both as regards extent and intricacy; the investigation of laws, subject not only to *fixed* modifications, but liable, in many instances, either to suspension or entire counteraction. We now begin to deal with living organs, not only requiring investigation, each in reference to itself, but, also, in reference to its connection with other parts. Who does not know that a full investigation of the laws of organization, so necessary to the dental surgeon, has ever required years of application of the greatest intellects, and the record of which has filled vast libraries, whereas, every principle connected with the mechanical portion of dentistry, may be enumerated on a single octavo page, and are, at least practically, within the reach of the most ignorant. Not only is there a wide difference between the physical, and organic laws, made by the greater number, and intricacy of the latter, but, perhaps, a still greater disparity grows out of the fact, that every rule of action applicable to living organized matter, is subject to the greatest modifications by extrinsic circumstances. For example, pure gold always melts at a given temperature; but as it is alloyed variously, the point of fusion changes; but it may be remarked, that for any change in composition, there is always an exactly corresponding change in the fusibility, its oxydability, &c. Therefore, the practical rules derived from these invariable principles are both few and simple. But let us examine, on the other hand, some of the principles governing living organic matter. There is, perhaps, no law more universal in its application, than that governing the natural healing process of wounds. Let an injury be inflicted upon any part of the body, for example, and there is an extraordinary flow of blood to the part, a greater or less inflammation supervenes, and an action is established calculated to heal the wound. This law is relied upon to restore all injuries, whether trivial or extensive, when no extrinsic circumstance obtains, to modify or counteract its legitimate operation and efficacy.

But while it is true, that the most serious injuries, unless upon vital organs, are, generally, thus speedily overcome; it is true, also, that a slight wound may lead to the most fatal consequences, by reason of modifying circumstances. While the most severe compound fracture may, in one case, hardly produce general irritation, or any constitutional effects, and the subject is rapidly restored to soundness, the scratch of a pin may, in another, prove suddenly fatal. Now, what constitutes this difference? Is not the same general law applicable to both? Notwithstanding the great difference in termination, this is clearly so; and the marked disparity in result has arisen entirely from the intervention of extrinsic circumstances, such as habits, constitution, &c. of the patient. The obstacles in the way of properly dealing with living organs are manifold, and the laws governing them, infinitely more difficult of comprehension, than when only physical or chemical principles are involved.

The following are some of the particulars upon which this proposition is based, viz. they are far more numerous, more difficult in themselves considered, they are subject to much greater modification by extrinsic circumstances, and what tends more than, perhaps, all other considerations to enhance the difficulty attending their control, is the fact, that every organ which is the subject of investigation, is intimately connected with, and may be under the control of all the others making up the corporeal frame.

We must see then, for the mere mechanic to be suddenly transported into this field of living organs, in which to act safely, he must possess a comprehensive knowledge of the organic laws, is a change which, to say the least, if he possess the slightest sensibility, must be exceedingly embarrassing. We think the blacksmith, or even the jeweller, must feel ill at ease in this new situation. Now it may be observed, that the change from mechanical to surgical dentistry, is precisely what we have described above, and skill in the one, is no greater guarantee for skill in the other, than success as a manufacturer of astronomical instruments, or of fixtures to restore broken limbs, an indication that the artisan must be an eminent astronomer or skilful surgeon.

If we need further illustration of the difficulty attending the operations upon, and the judicious management of, the living teeth, deemed so simple by the multitude, we have an ample one in the *failures*, which have so commonly, nay, almost universally attended them. Nor have the miseries of this malpractice been limited to mere failures, to accomplish what was promised, but the already existing evils have often been so much enhanced, as to leave, in the minds of thousands, only disgust for the act, and contempt for the whole fraternity of its practitioners. Such is the state of public opinion, thus created, even at the present day, that we venture to assert, that by far the greater proportion of the inhabitants of the United States, look upon dental operations, as only evil devices, to gull the unsuspecting, while many more entertain serious doubts whether they possess any intrinsic worth. But we claim that all these evil results have been exclusively the offsprings of abuses of dental practice, and are in no way necessarily connected with it. No proposition has been more fully demonstrated than that *judicious* dental operations, have, generally, perfectly controlled, or averted the diseases incident to the teeth and mouth, and their effects upon the general system. But it must be remembered that these beneficial results are confined to the practice of ***intelligent, honest and skilful operators***, who, we are sorry to add, are far too few, compared with the great number who presume to call themselves dentists, and assume the responsibilities of this difficult and important art.

But shall we ascribe the lack of success of this large majority, *solely* to wilful dishonesty, and a base design to fill their own pockets at the expense of the comfort, and, perhaps, the lives of their fellow men? Although there may be, and, doubtless, are some among them so depraved, yet, it is but charity to presume, that many, and we believe most of those, who are empirical, both in their views and practice, are honestly so. In other words they commenced practice without any adequate notions of the amount, or kind of knowledge required, or even of what constitutes good operations. In this way they proceed, till some benevolent (?) parson, or M. D., who wished to secure *cheapness*, volunteers to certify, that his operations are of the first order!! He now, thinking his prospect of success complete, jogs, along, perhaps, for years, never doubting that his own skill will eventually secure him fame and fortune. And, although, his cases of failure far out number his successful ones, yet he looks upon them, as a necessary consequence, and attributes them all to a lack of virtue in the art. Now the difficulty lays in an unsuspected quarter, in the fact that he commenced and has remained in such profound ignorance of the whole ground, that he had never discovered the extent of the difficulties, or dangers, in the way of success, and, of course, had made no proper effort to remove them.

It is said that the operation for lithotomy was first performed by an ignorant monk, who knew nothing of the relative situation or importance of the parts it involved. His ignorance was such, that he fearlessly plunged in his knife, and was occasionally successful. After surgery was cultivated as a science, and this operation, one of the most difficult even to men of the greatest skill, came to be fully understood, and performed on scientific principles, this monk was persuaded to study the anatomy of the parts involved, and, hence, to realise its difficulties, and its dangers. The result was, that he could never be again induced to attempt it.

We have no doubt that a similar result would follow, if many of our dentists, were to be instructed more fully in the importance and difficulties involved in many of the operations connected with dental surgery. Did our space permit, we would proceed to point out by referring to these various operations, and the successive steps in them, the demands for skill, and scientific intelligence, which they respectively make upon all who presume to perform them, but this we shall leave for a future occasion, only adding our full conviction that many, if not most of the evils of quackery, have, doubtless, originated in a false estimate, on the part, no less of the operators, than of the public, of the kind, and amount of knowledge, required, and of the comparative skill involved in the mechanical and surgical department. We are fully persuaded that just so long as window specimens of plate work, are set up, either in the estimation of the public or dentists themselves, as an index of proper qualification for the practice of dental surgery, just so long will the infinitely more important, and difficult, operations upon the natural teeth, be neglected and abused, and quackery flourish, and abound. On the other hand, just so soon as the *real* index of qualification is clearly defined, and understood, by all, quackery will die a natural death; will cease to exist for want of support, and thousands who are now "rioting in the ill-gotten fruit of unblushing impudence and empiricism," would be compelled to seek some employment less intimately connected with the comfort of the race. While many would thus be *forced* from the track, others by being made aware of the real obstacles to success, would either voluntarily withdraw, or, by proper effort, come up to the true standard, and many of these, we have no doubt, would become useful and honorable members of the profession.

Next, let us inquire how far the physician, receiving only a medical education, is prepared to assume the practice of either class of dental operations. Our views upon this topic have been already fully expressed in the Journal, upon another occasion, although not in precisely this connection. While we have ever advocated the propriety, and, indeed, necessity of medical knowledge, as "*preparatory* to the pursuit of dental surgery," we have never, for a moment, considered even a thorough medical education, as qualifying a student for operative dentistry. A good knowledge of arithmetic is essential to a practical engineer, yet, who would not see the absurdity of employing an arithmetician simply, to survey a tract of land where accuracy was of moment, or to grade for the construction of a rail-road, where capital was to be invested. An acquaintance with mathematical instruments would be by no means expected of such an one, much less would he be presumed capable of applying them in practice. The most ignorant would startle at such presumption. But this would be just as rational as to expect from a physician, that kind of practical skill, so desirable as a surgeon dentist. It is true that he has, or ought to have, learned, the *principles* upon which the diseases of the teeth and mouth are treated, but where has he learned specific directions, and by whom has he had his attention specially directed to these diseases. We venture to assert, that it is neither from medical books, nor by medical teachers. Experience fully corroborates this conclusion. We have seen, for example, in numerous instances, physicians, who were suffering from the accumulation of tartar about their teeth, who were greatly surprised to learn, that it was a foreign substance; and when a piece of it was removed, with an air of serious concern, inquired, if it was not a piece of the tooth.

When we hear, as we often do, the opinion of some physician, that a brush should never be used in the mouth, through fear of destroying the enamel, and find a whole community following his sage advice, we confess it calculated to excite our combativeness, rather than veneration for his wisdom, at least as a dental adviser.

Again, when we see our M. D's. pouring into the stomachs of their "*nervous* patients," the rinsings of an entire apothecary shop, and find that they never have even thought to inquire into the state of the mouth and teeth, it excites a contempt, only surpassed by a feeling of pity for their patients.

When, moreover, we are taught by those who have been termed "the lights of medical science," to believe that caries in the teeth proceeds from causes wholly beyond the control of man, and as a conclusion, all effort to preserve these useful organs must be unavailing, we are constrained to feel that something more than medical skill is required to constitute a safe dentist. We might proceed to enumerate every fundamental principle constituting the basis of correct dental theory and practice, and we should find the opinions entertained of them by physicians, and by the public, who have borrowed these opinions directly from this source, to accord as little with truth and safety, as they have in the examples already given. Had we no other proof that their attention has never been properly directed to the preservation of the teeth, and the well-being of the mouth, we have an ample one in their reckless administrations of acid medicines, without any conservative or counteracting means being employed.

By the course of acid tonics which are carelessly fed to convalescing patients, thousands have their teeth irreparably ruined. Well is it, for such physicians, that they have a "scape goat," at hand, *calomel*, poor *calomel*, as a willing agent, is ready to shoulder the sin and bear it into nonentity. Thus patients go through life cursing calomel, for what they might justly offset against the long bill of their doctor.

Another topic which is deserving attention in this connection, is, the awkward and unscientific manner in which teeth are extracted by most physicians. This is a subject which is the more to be noticed, as it is one which they *profess* to understand, and daily practice, in which ignorance, hence, is absolutely culpable. But how stands the fact? To say nothing of their fit-out of instruments, which generally consists of a rusty turnkey, wound with a pocket handkerchief, and a jack-knife; the careless manner in which the operation is performed, is, by no means, such as should characterise a skilful dentist. How often is the wrong tooth lugged out in connection, it may be, with the one aimed at, by a mal-adjustment of the instrument, or perhaps, for want of discrimination, which both physician and patient had failed to make. Two cases of the latter kind, have fallen under our own observation, within the last three months. In one, a gentleman, from a neighboring village, called on us, and wished us to extract one of his cuspidati of the upper jaw, observing that two attempts had been made to do so, *that day*, by the physician of his place. On examining the tooth, we could not find the slightest decay in it, except a small point, on the external surface, near the gum. This being sensitive to the touch, had led both patient and doctor to the conclusion that the nerve must be exposed, and therefore it should be extracted!! On examining it, we assured him that *this* tooth could not have been the cause of the great pain he had suffered, and that we must look farther for the real origin, which we soon decided to be in the lower dens sapientiæ of the same side, which was exceedingly decayed, and the nerve exposed. Although he could hardly be induced to believe it the source of his pain, yet consented to have it removed. He called on us the next day, thanking us heartily for the relief he had thus obtained, and rejoicing in the ill-success of the physician, in his attempt to rob him of one of his most valuable teeth, leaving *undisturbed*, the cause of all his troubles.

In the other case, a perfectly sound and healthy front lower incisor, was actually removed with a view to alleviate pain, caused by a tooth equally distant from the one sacrificed, as in the case first described.

From such examples as these, we feel that we are doing no injustice to physicians, if we do not award to them the skill necessary to a judicious practice of dental surgery. Such cases not only discover a most serious defect in medical education, but they call loudly for a remedy. This, to physicians, must come through the medium of our medical schools.

These institutions must be regarded exceedingly imperfect without a department *expressly* supplying what must be looked upon a most radical deficiency in the education of strictly a *medical practitioner*, much more, if he would amalgamate with it, dental practice.

Another argument in favor of the position, that a physician is no dentist, is found in the fact, that they have ever been as ready dupes to dental quackery, as are to be found in any class of society. Nothing is more common than to hear learned doctors bewailing their toothless lot, and ascribing the whole to the imposition of some *travelling* empiric, calling himself a dentist. Now could a man, sufficiently enlightened upon dental science, and practice, to be *himself* a safe adviser, and operator, be thus duped? Would such an one consent to become a willing subject on which to have displayed the magic virtues of a mercurial paste, with hope to save a favorite tooth? Such misfortunes, and moans, from medical men, we confess, have never inordinately excluded our sympathy, especially if we learn in the connection, that they too, have "a snug little box of tools," in which they keep, for the benefit of such as desire dentistry as well as physic, a few rolls of ready prepared tinfoil, a paper of silver filings, a small vial of mercury, and what is absolutely indispensable, a plenty of kreosote and arsenic.

If it still be urged that the physician may be easily transformed into a dentist, we would again inquire whence is his special knowledge of the science which will enable him to recognise, and successfully treat the various dental diseases? Where has he acquired that dexterity of hand, which it has cost the most ingenious, years of youthful training to command? By what law of intuition is he enabled to select and rightly apply the instruments best adapted to each operation? Where, in short, has he learned to overcome the thousand difficulties, which are met at every turn, by the most intelligent and experienced practitioner? If such inquiries confound him, as they certainly must, if he relies solely upon his medical acquirements, to loose his tongue, we would advise him for his own sake, and for the sake of humanity, to abandon this unmerciful project of destroying his neighbor's teeth, either for the few shillings which he may thus acquire, or for the sake of husbanding his odds and ends of time.

Should this course find him in the possession of a surplus of leisure, which he regards burdensome, we would advise him to consult his *library* for relief.

